# Supplementary material for: The clade-specific target recognition mechanisms of plant RISCs
Source: Nucleic Acids Res. 2024 Apr 16;52(11):6662–73. doi: 10.1093/nar/gkae257 (PMC11194062; doi:10.1093/nar/gkae257)
Supplement: gkae257_Supplemental_File [file gkae257_supplemental_file.pdf]

## Supplementary information

```
                21U-guide
21U duplex      pUGAGGUAGAUCCUUGUAUAGU(M)
                  ||| |||| |||||||||
             (M)UCUCUCGAUCUUGGAACAUAUp

                21A-guide
21A duplex      pAGAGGUAGAUCCUUGUAUAGU(M)
                  ||| |||| |||||||||
             (M)UCCCUCGAUCUUGGAACAUAUp

                24A-guide
24A duplex      pAGAGGUAGAUCCUUGUAUAGUCUC(M)
                 |||| ||| |||||| ||||||
             (M)UCUCUCUAUCAAGGAACUUAUCAGp
```

**Supplementary Figure S1. Small RNA duplexes used in this study.**

The 3′ end of small RNA is modified with 2′-O-methyl (M).

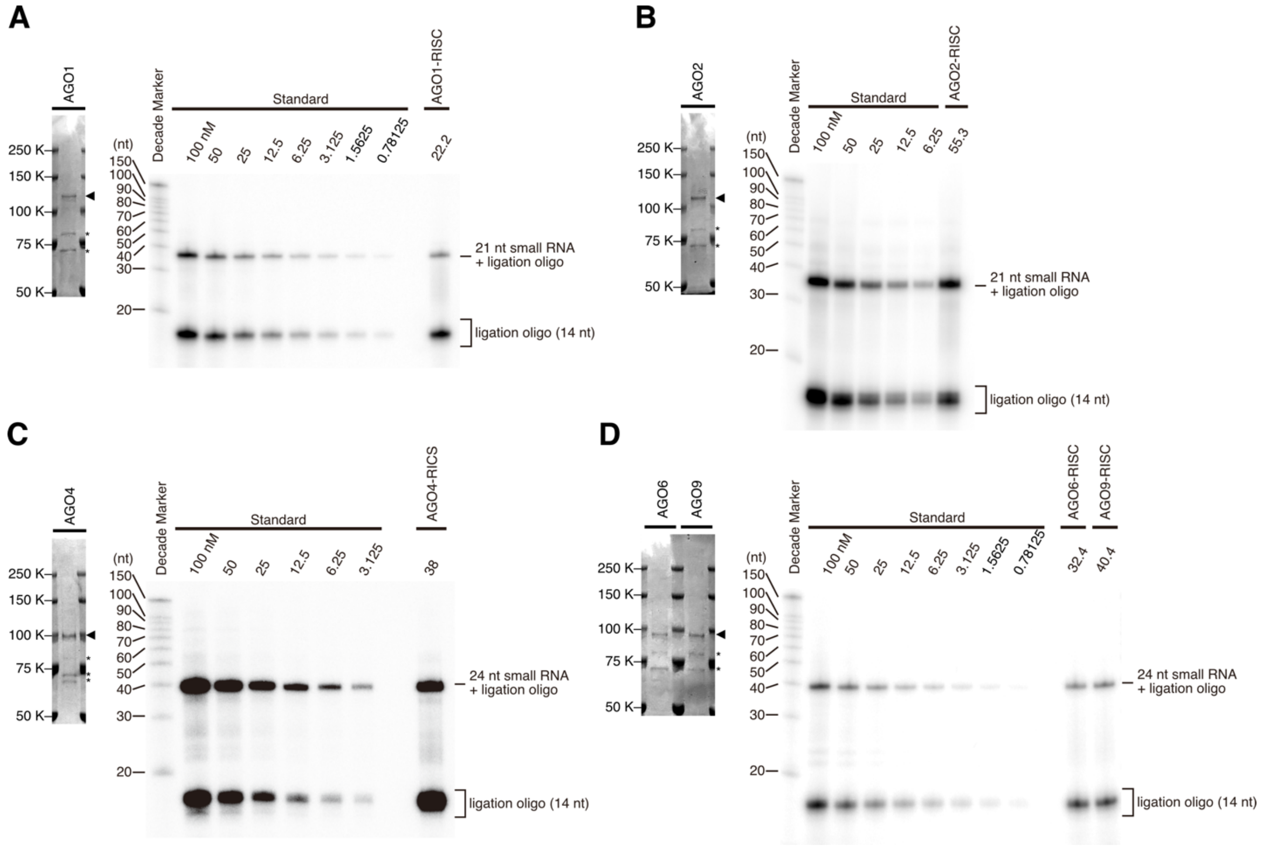


**Supplementary Figure S2. Quantification of RISC using the splint ligation method.**

A. AGO1-RISC, B. AGO2-RISC, C. AGO4-RISC, D. AGO6- and AGO9-RISCs. (left) Quality of recombinant AGOs. Arrowheads denote AGO proteins and asterisks indicate co-purified proteins. (right) After deproteinization of the purified RISC, the guide strand and a radiolabeled ligation oligo were brought into proximity using a bridge oligo, followed by ligation (21/24-nt small RNA + ligation oligo). Unreacted ligation oligo, which was not completely dephosphorylated by alkaline phosphatase, is observed at the lower part of the gel. A known concentration of small RNA was subjected to the same reaction, and serial dilutions were performed to create a standard curve. Decade Marker (Thermo Fisher Scientific) was used as a size marker for RNA.

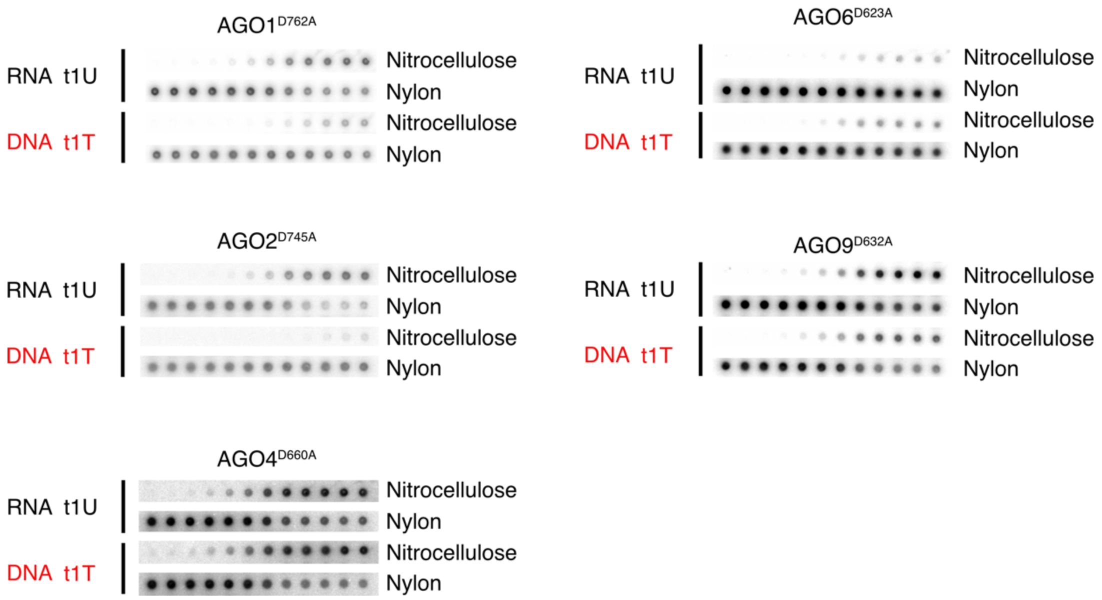


**Supplementary Figure S3. Equilibrium binding assay of t1U/T to RISC.**

RISC-bound target is blotted onto the nitrocellulose membrane and unbound target RNA is blotted onto the nylon membrane.

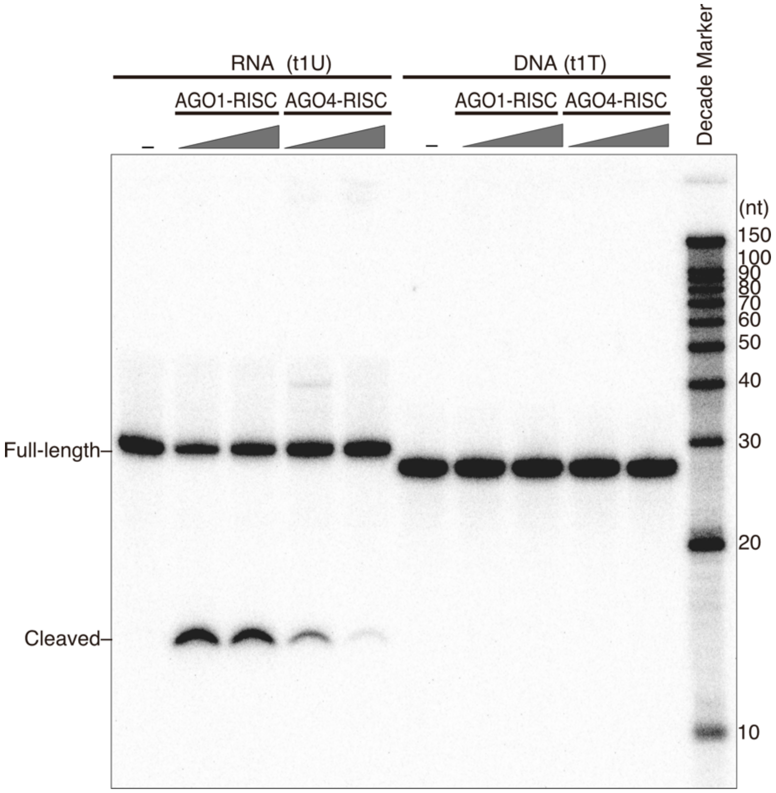


**Supplementary Figure S4. Cleavage assays of t1U and t1T by wild-type AGO1- and AGO4-RISCs at different concentrations.**

Wild-type AGO1- and AGO4-RISCs cleaved RNA (t1U) but not DNA (t1T). Decade Marker (Thermo Fisher Scientific) was used as a size marker for RNA.

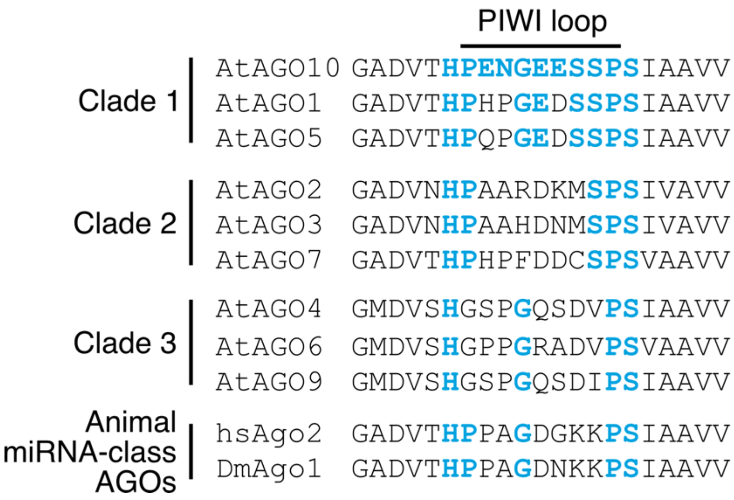


**Supplementary Figure S5. Multiple alignment of amino acids in the PIWI loop region in *Arabidopsis* AGOs and animal AGOs.**

Bold blue letters indicate amino acids identical to the PIWI loop in AGO10.

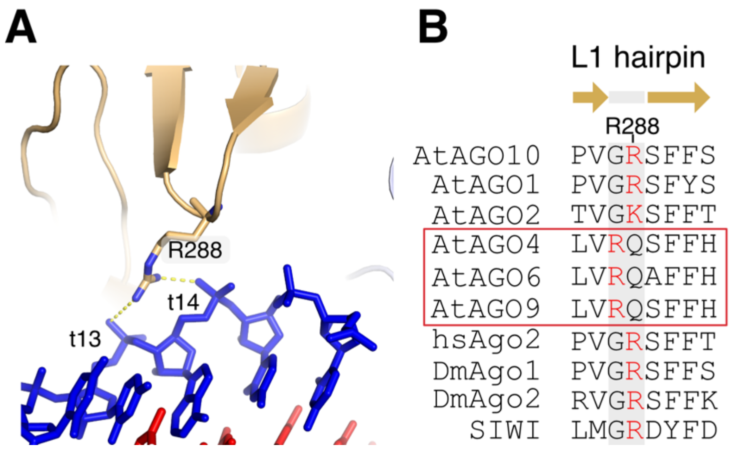


**Supplementary Figure S6. The L1 hairpin contacts the target RNA in the 3′ supplementary region.**

A. The structure of L1 hairpin in the AGO10-miRNA-target complex [PDB ID: 7SWF]. The arginine residue in the L1 hairpin contacts the backbone of t13 and t14 of the target RNA.

B. Multiple alignments of the amino acid sequence of the L1 hairpin. The turn of the hairpin is formed by different amino acids only in clade 3 AGOs.
